# The Effect of the Capsular Bag on the Optical Performance of an IOL Measured in an Ex Vivo Model

**DOI:** 10.1167/iovs.67.3.46

**Published:** 2026-03-19

**Authors:** Margarita Karaivanova, Grzegorz Łabuz, Agnieszka Zielińska, Zhiyi Wu, Leoni Britz, Sabrina Wohlfart, Philipp Uhl, Gerd Uwe Auffarth, Maximilian Hammer

**Affiliations:** 1 Heidelberg University Hospital, Department of Ophthalmology, Heidelberg, Germany; 2 David J. Apple Laboratory for Vision Research, Heidelberg, Germany; 3 Institute of Physics, Faculty of Physics, Astronomy and Informatics, Nicolaus Copernicus University in Toruń, Toruń, Poland; 4 Eye Center, The Second Affiliated Hospital, Zhejiang University, Hangzhou, People's Republic of China; 5 Institute of Pharmacy and Molecular Biotechnology, Heidelberg, Germany; 6 Faculty of Biosciences, Heidelberg University, Heidelberg, Germany

**Keywords:** intraocular lens (IOL), capsular bag, optical quality, modulation transfer function, simulated visual acuity (SimVA), photic phenomena, forward light scattering

## Abstract

**Purpose:**

The purpose of this study was to investigate the effect of the capsular bag on the optical performance of different intraocular lens (IOL) designs, and to develop and validate an ex vivo human capsular bag model that enables quantitative optical evaluation under anatomically relevant biomechanical conditions.

**Methods:**

Human cadaver eyes (*n* = 8) were prepared using a modified Choi-Apple technique to isolate the intact uveolenticular complex, which was stabilized with custom-designed 3D-printed rings. Following lens extraction, five commercially available IOLs (1 monofocal-plus, 2 Extended Depth of Focus [EDoF], and 2 trifocal designs) were sequentially implanted. Optical performance was evaluated with the OptiSpheric IOL PRO2 and a modified C-Quant setup. Metrics included modulation transfer function (MTF), simulated visual acuity (SimVA), point spread function (PSF), and forward light scattering (FLS).

**Results:**

The model enabled optical assessment of IOLs within the biomechanical environment of the capsular bag while capturing biologically relevant inter-specimen variability. Baseline straylight from phakic capsular bags averaged 0.75 ± 0.21 log(s), comparable to values in young healthy eyes. Defocus curves and United States Air Force (USAF) resolution images revealed IOL-specific variations consistent with reported clinical outcomes. Diffractive EDoF and trifocal IOLs exhibited higher susceptibility to capsular irregularities and more pronounced photic phenomena compared with monofocal-plus and refractive EDoF designs.

**Conclusions:**

This study provides a platform for quantitative evaluation of the optical quality of IOLs within explanted human capsular bags, using the surrogate parameter SimVA. This approach enables assessment of lens optical performance within its designated anatomic setting, offering valuable predictive insights into optical quality and visual outcomes before initiating clinical trials.

Annually, more than 7 million cataract surgeries are conducted in Europe and over 20 million worldwide.^1^ This high surgical volume has driven continuous innovation in intraocular lens (IOL) design, with new models and technologies being introduced each year to optimize visual outcomes and meet the growing demands of an aging population.^2^ These include multifocal, extended depth-of-focus (EDoF), and accommodating IOL with complex optical profiles, promising to achieve spectacle independence for both near and distance vision and to enhance postoperative patient satisfaction.^3^^–^^5^

However, the preclinical evaluation of these new models remains largely limited to optical bench setups, such as International Organization for Standardization (ISO)-standardized model eyes and optical metrology systems.^6^^–^^9^ Although these platforms provide valuable information on lens performance in idealized conditions, they do not account for the complex anatomic and biomechanical environment of the human eye – especially the influence of the capsular bag, which plays a critical role in the positioning, tilt, decentration, and dynamic behavior of the IOL over time.^10^^–^^12^ To date, there are no standardized preclinical methods that adequately assess the interaction between IOLs and the capsular bag in a way that captures the resulting optical performance.

Over the past decades, several ex vivo capsular bag models have been developed to examine the proliferation of residual lens epithelial cells (LECs) in culture following a simulated cataract surgery.^13^^–^^18^ Whereas these models have proven valuable for investigating cellular behavior and posterior capsule opacification, they are primarily tailored for microscopic analyses and are not able to support precise optical assessment of the IOL within the native geometry of the capsular bag. As a result, the influence of the capsular bag and its dynamic interactions with implanted optics remains an underexplored aspect of IOL performance.

To bridge this gap, we aimed to develop an ex vivo model that allows the optical evaluation of IOL implanted into extracted human capsular bags. Our approach builds on the Choi-Apple View system – a lenticular imaging technique, introduced by Son et al.^19^ – by integrating it into an advanced setup that permits quantitative analyses of IOL optics within the biomechanical environment of the capsular bag. This model enables complex optical measurements — including modulation transfer function (MTF), point spread function (PSF), and forward light scattering (FLS), already established as valuable parameters for testing the visual performance of complex intraocular devices.^7^^,^^20^^–^^22^ To first characterize this model, we selected and evaluated five IOLs representing a range of contemporary optical designs commonly used in clinical practice. These included a monofocal-plus IOL (Tecnis Eyhance ICB00), two EDoF IOL (AcrySof Vivity, Tecnis Symfony), and two trifocal IOLs (Tecnis Synergy, AcrySof PanOptix).

## Methods

### Human Cadaver Eyes

Human phakic eyes (*n* = 8) with intact uvea and lens structures were obtained from 6 donors (range = 53–82 years, mean age - 64.33 ± 9.99 years, 3 men and 3 women) within 24 hours postmortem through the Eye Bank of the Heidelberg University. For the use of human tissue, ethical approval was gathered prior to the study initiation from the local ethics committee of the Heidelberg University (approval number: S-134/2018). All procedures have been carried out in accordance with the tenets of the Declaration of Helsinki for the use of human tissue as well as the guidelines of the ARVO Best Practices for Using Human Eye Tissue in Research. Supplementary Table S1 presents the characteristics of human tissue used in the study.

### Tested Intraocular Lens Designs

Five different commercially available IOLs were evaluated in this study (Table 1). Each IOL was assessed under standardized experimental conditions to compare its optical performance and functional properties. All IOLs had a nominal power of +20 diopters (D).

**Table 1.** Details on the Evaluated IOLs

Name	Manufacturer	Classification	Optical Design
Tecnis Eyhance ICB00	Johnson & Johnson Vision, Irvine, CA, USA	Monofocal Plus IOL	Refractive
AcrySof IQ Vivity	Alcon, Fort Worth, TX, USA	Extended Depth of Focus (EDoF) IOL	Refractive
Tecnis Symfony	Johnson & Johnson Vision, Irvine, CA, USA	Extended Depth of Focus (EDoF) IOL	Diffractive
Tecnis Synergy	Johnson & Johnson Vision, Irvine, CA, USA	Trifocal IOL	Diffractive
AcrySof IQ PanOptix	Alcon, Fort Worth, TX, USA	Trifocal IOL	Diffractive

### Surgical Preparation

**Figure 1.**
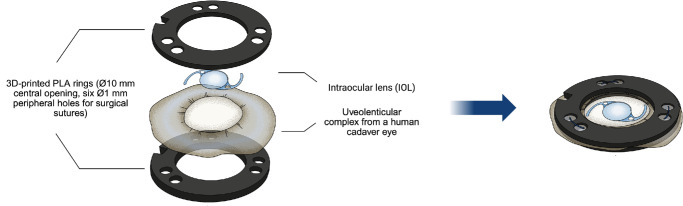
Design of the capsular bag model. The model consists of two custom-designed 3D-printed rings, between which the uveolenticular complex is positioned and held in place. The rings are secured together using surgical sutures, thereby stabilizing the capsular bag for experimental use.

Human cadaver eyes were prepared using a modified Choi-Apple technique. First, an equatorial sclerotomy of the bulbus was performed, followed by vitrectomy. The chorioretinal tissue was then carefully separated from the sclera via hydrodissection. Once the uveolenticular complex – comprising the choroid, ciliary body, and lens capsule – was fully exposed and detached from the scleral spur, it was placed on a custom-designed 3D-printed ring with a central hole of 10 mm in diameter, as shown in Figure 1. This ensures that the capsular bag remains freely suspended, protruding through the hole, supported solely by zonular tension, as in vivo. A second ring of the same size and with an identical central hole was placed on top of the uveolenticular complex. Both rings, with the uveolenticular complex between them, were sutured together through six 1-mm peripheral holes using surgical sutures (Resolon 5-0, Resorba).

Subsequently, an iridectomy is performed, followed by an anterior capsulorhexis. The natural lens is then extracted via hydroexpression, and any residual cortical lens fibers are removed through using irrigation/aspiration (Geuder S3, Geuder AG, Heidelberg, Germany).

### Optical Bench Setup

**Figure 2.**
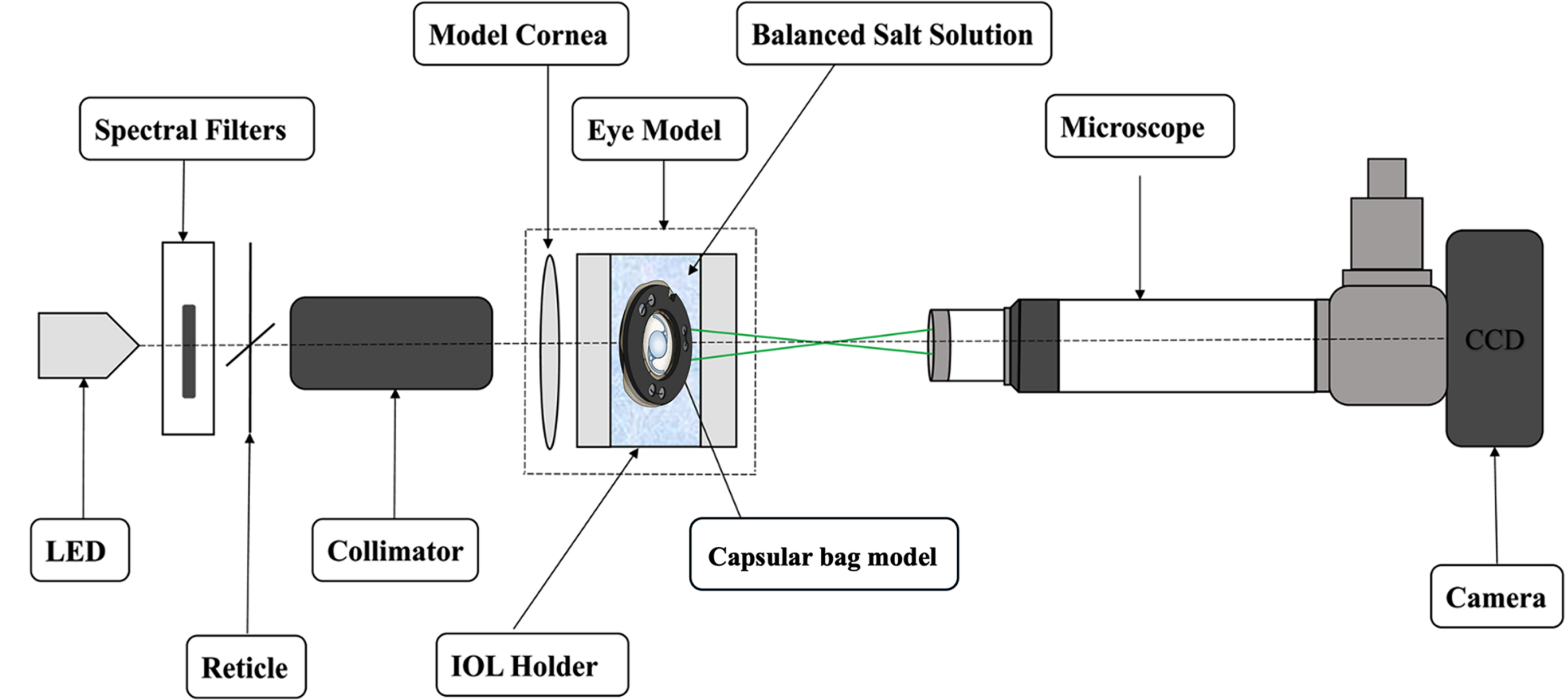
Schematic illustration of the optical-metrology setup. CCD, charge-coupled device; LED, light-emitting diode. Figure modified with permission from Łabuz et al.^26^

The optical properties of the ex vivo capsular bag model were assessed using the OptiSpheric IOL PRO2 (Trioptics GmbH, Wedel, Germany), a high-precision,^23^ semi-automated optical testing device (Fig. 2), which has been established as a reliable method for evaluating IOLs across various optical designs.^24^

Optical quality was analyzed based on the MTF – a widely used metric in both IOL research and industry for assessing lens performance and efficiency.^7^^,^^21^^,^^22^^,^^25^^,^^26^ The MTF is an objective parameter that quantifies an optical system's ability to reproduce the contrast of a grid pattern across different spatial frequencies, analogous to how contrast sensitivity is evaluated in clinical testing. The MTF was calculated over a range of spatial frequencies extending beyond the optical system's cutoff frequency. For practical reasons, MTF curves are reported up to 100-line pairs per millimeter (lp/mm), corresponding approximately to a visual acuity of 20/20. The MTF value at 50 lp/mm (approximately equivalent to a visual acuity of 20/40) was extracted and used as a representative metric for evaluating the optical performance of the studied lenses, as it is commonly used as a comparative criterion in optical bench studies. Measurements were obtained in both polychromatic light and monochromatic green light (546 nm), using apertures of 3.0 mm and 4.5 mm, corresponding to pupil sizes commonly found in patients over 60 years under photopic and mesopic conditions.^27^ A cornea model featuring the natural level of spherical aberration was also applied.^28^ The area under the MTF curve (MTFa) was measured over a defocus range from +1.5 D to –4.0 D at the spectacle plane to estimate the simulated visual acuity (SimVA), following the method described by Alarcon et al.^25^ and later validated by Vega et al.^22^ The SimVA was calculated based on the ANSI Z80.35-2018 recommendations as follows:

$$\mathrm{VA}=a\cdot \mathrm{MTF}a^{b}+c$$

where a = 0.085, b = −1.0, and c = −0.21, and the MTFa is the calculated area under the measured MTF curve obtained in polychromatic light.

This approach provides a reliable surrogate for predicting in-vivo visual performance in an optical bench setting. Besides, the 1951 United States Air Force (USAF) resolution test chart was recorded to confirm the optical performance qualitatively.

Beyond providing insights into visual acuity and contrast sensitivity, the optical bench setup also aids in predicting the likelihood of photic phenomena. In in vitro studies, this is often assessed using the PSF, which models how light is distributed within an optical system, simulating real-world visual experiences – such as the perception of headlights while driving at night.^29^^–^^32^ This test is particularly useful for detecting stray light that does not contribute to retinal image formation but instead causes visual disturbances, including halos, glare, and starbursts.^33^ A polychromatic PSF, that is, an image of a 0.1-mm pinhole, captured through a 4.5 mm aperture, was used to compare the light distribution beyond the center of the different IOL models.

### Forward Light Scattering Setup

To quantify forward light scattering, we used a technique originally introduced by van den Berg et al.^34^ for the clinical assessment of ocular straylight. This methodology was later adapted by Łabuz et al.^20^ for in vitro evaluation of IOLs and has since been implemented for optical analysis of various intraocular substances.^35^^,^^36^

**Figure 3.**
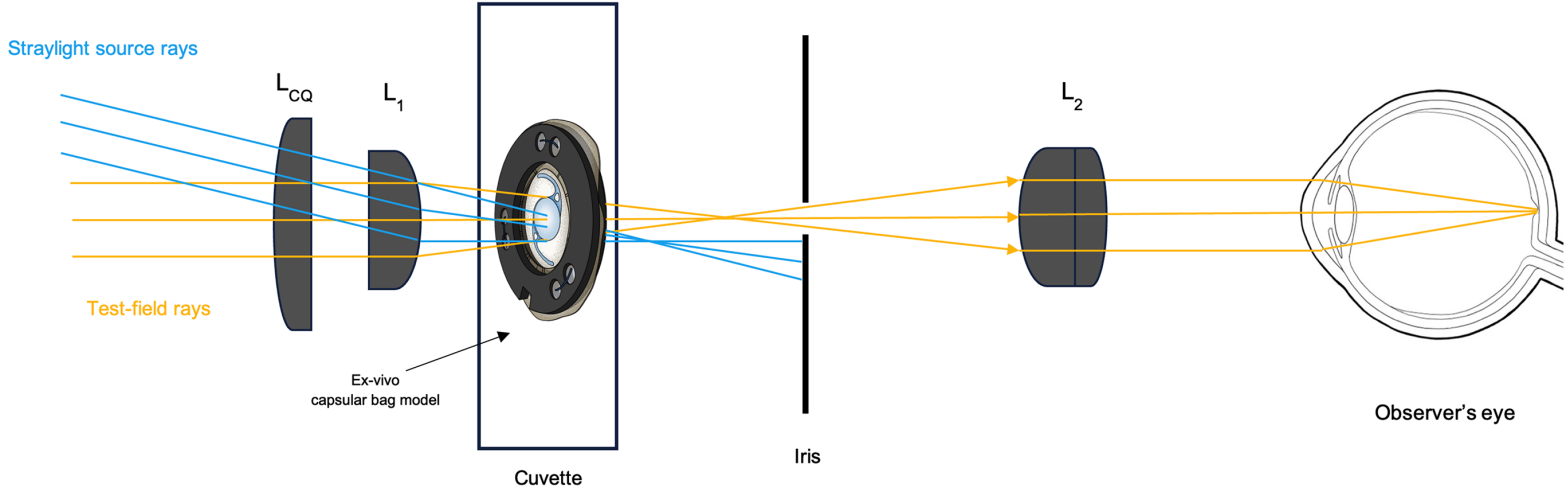
Schematic illustration of the C-Quant adaptation used to objectively measure forward light scattering in vitro. Figure modified with permission from Hammer et al.^36^

A commercially available diagnostic device, the C-Quant (Oculus, Wetzlar, Germany), was modified by mounting a custom-designed optical setup, which ensures that only the sample is exposed to the straylight source, whereas eliminating any contribution from the observer's eye through a field-stop mechanism. As a result, the observer can evaluate the light diffused by the object without any interference caused by the observer's own ocular straylight, which allows a consistent and unbiased assessment. Straylight is expressed as the logarithm of the straylight parameter “s”, or log(s). Figure 3 illustrates the concept of in vitro straylight measurement. More details on this methodology are presented both in Łabuz et al.^20^ and Hammer et al.^36^

### 3D-Printing Setup

The custom-designed components for the capsular bag model were created using FreeCAD (FreeCAD Community, open-source software) and 3D-printed with the Creality K1 MAX 3D printer (Creality, Shenzhen, People's Republic of China).

### Measurement Protocol

First, all 5 IOLs were hydrated in deionized water at room temperature for 24 hours. Baseline measurements of MTF and PSF were recorded for each IOL before implantation. After preparing and securing the capsular bag within the model, baseline optical measurements (forward light scattering, MTF, and PSF) of the capsular bag and the corneal lens were also obtained.

**Figure 4.**
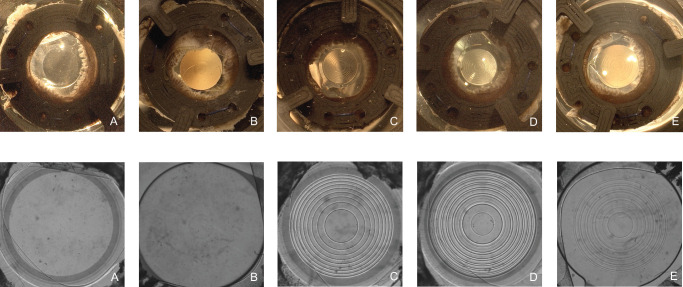
Images of the five IOL designs inside the capsular bag model, captured with light microscopy (*above*) and with the charge-coupled device (CCD) camera of the OptiSpheric IOL PRO2 (*below*). (**A**) Tecnis Eyhance ICB00. (**B**) AcrySof IQ Vivity. (**C**) Tecnis Symfony. (**D**) Tecnis Synergy. (**E**) AcrySof IQ PanOptix.

Each IOL was subsequently implanted into the capsular bag. Following implantation, light microscopy and optical measurements of the IOL within the capsular bag were obtained before the IOL was explanted (Fig. 4). Prior to implanting the next IOL, the capsular bag was thoroughly examined for any anatomic defects. This process was repeated for all capsular bags, with the five IOLs implanted in a randomized order for each capsular bag to ensure an unbiased assessment. The specific capsular bag-IOL combinations included in the analysis are detailed in Supplementary Table S2.

## Results

### Baseline Optical Properties of the Capsular Bag

**Figure 5.**
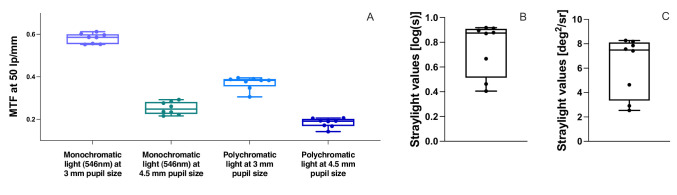
Baseline measurements of the capsular bag. (**A**) MTF values at 50 lp/mm for the capsular bag at 3 mm aperture and 4.5 mm aperture measured under monochromatic (546 nm) and polychromatic conditions. (**B**) Straylight values of the capsular bag in [log(s)]. (**C**) Straylight values of the capsular bag in [deg^2^/sr].

Baseline measurements prior to IOL implantation are shown in Figure 5. The mean MTF at 3.0 mm pupil size at a spatial frequency of 50-lp/mm was 0.58 ± 0.02 [range: 0.55, 0.61] under monochromatic illumination and 0.37 ± 0.03 [0.31, 0.40] under polychromatic illumination. At 4.5 mm pupil size, mean MTF values decreased to 0.25 ± 0.03 [0.22, 0.29] and 0.18 ± 0.02 [0.14, 0.21], respectively. Straylight of the native capsular bag was 0.75 ± 0.21 [0.41, 0.92] log(s), corresponding to 6.17 ± 2.42 [2.54, 8.27] deg^2^/sr in linear units.

### MTF Metrics of IOLs Implanted in the Capsular Bag Compared to Control Measurements of the IOLs

**Figure 6.**
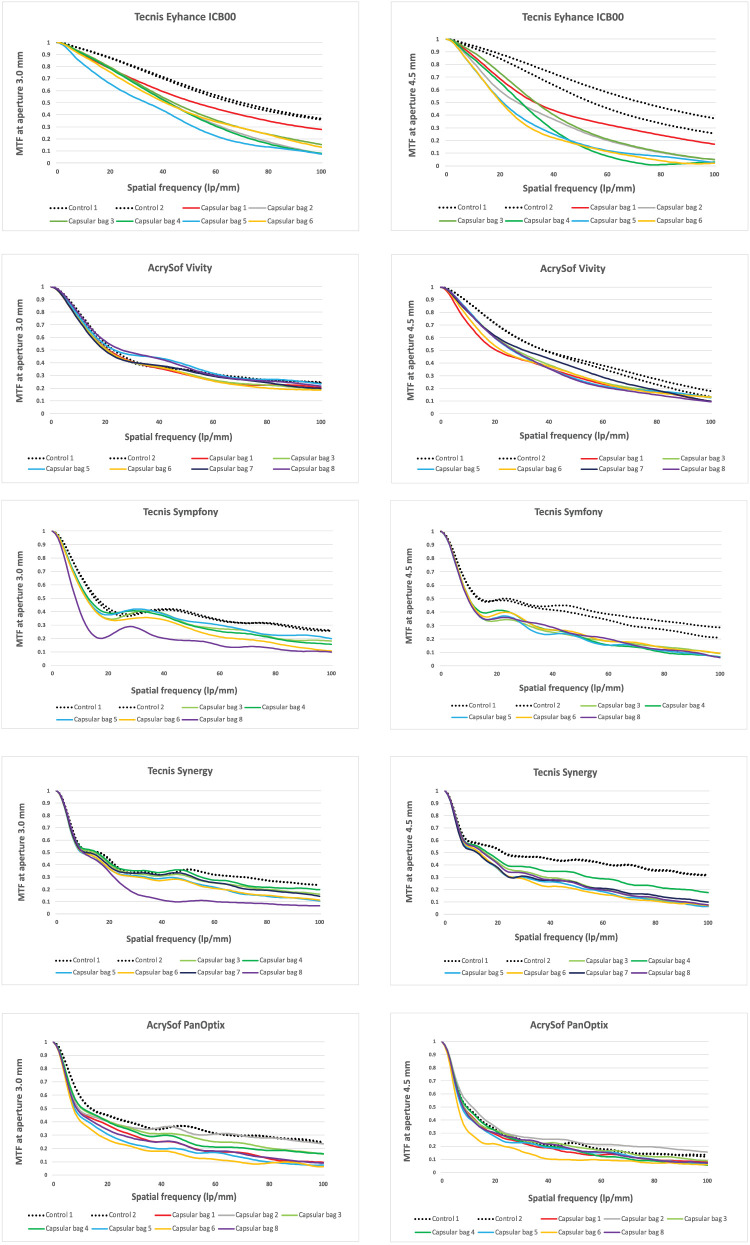
MTF at best far focus across spatial frequencies (0–100 lp/mm). The *solid lines* represent the IOL measured within the capsular bags, whereas the *dashed lines* show the same IOL measured prior to implantation.

The comparison between control and post-implantation MTF values at the best far focus under monochromatic illumination is summarized in Figure 6.

At a 3.0 mm pupil, Tecnis Eyhance implanted in the capsular bag achieved a mean MTF value across all capsular bags of 0.42 ± 0.06 [0.32, 0.52] at 50 lp/mm, representing a 1.5-fold decrease compared to the control (0.62). AcrySof Vivity maintained performance with an MTF of 0.34 ± 0.03 [0.31, 0.38], showing no reduction relative to control (0.34). Tecnis Symfony yielded 0.27 ± 0.05 [0.18, 0.33], a 1.4-fold decrease versus control (0.39), while Tecnis Synergy demonstrated 0.27 ± 0.09 [0.10, 0.34], corresponding to a 1.3-fold decrease versus control (0.36). AcrySof PanOptix showed 0.23 ± 0.06 [0.14, 0.32], with a 1.6-fold decrease compared to control (0.36).

At a 4.5 mm pupil, differences between implanted and control IOLs were more pronounced. Tecnis Eyhance measured 0.24 ± 0.09 [0.16, 0.37] at 50 lp/mm, a 2.4-fold decrease compared to control (0.59). AcrySof Vivity showed an MTF of 0.30 ± 0.03 [0.27, 0.36], a 1.4-fold decrease from control (0.42). Tecnis Symfony achieved 0.22 ± 0.01 [0.21, 0.24], a 1.8-fold decrease relative to control (0.40). Tecnis Synergy performed similarly with 0.25 ± 0.04 [0.20, 0.33], also a 1.8-fold decrease compared to control (0.44). AcrySof PanOptix showed 0.17 ± 0.04 [0.10, 0.24], and thus the smallest relative decline, corresponding to only a 1.2-fold decrease versus control (0.22).

### Simulated Visual Acuity Curves Derived From MTFa Under Polychromatic Illumination

**Figure 7.**
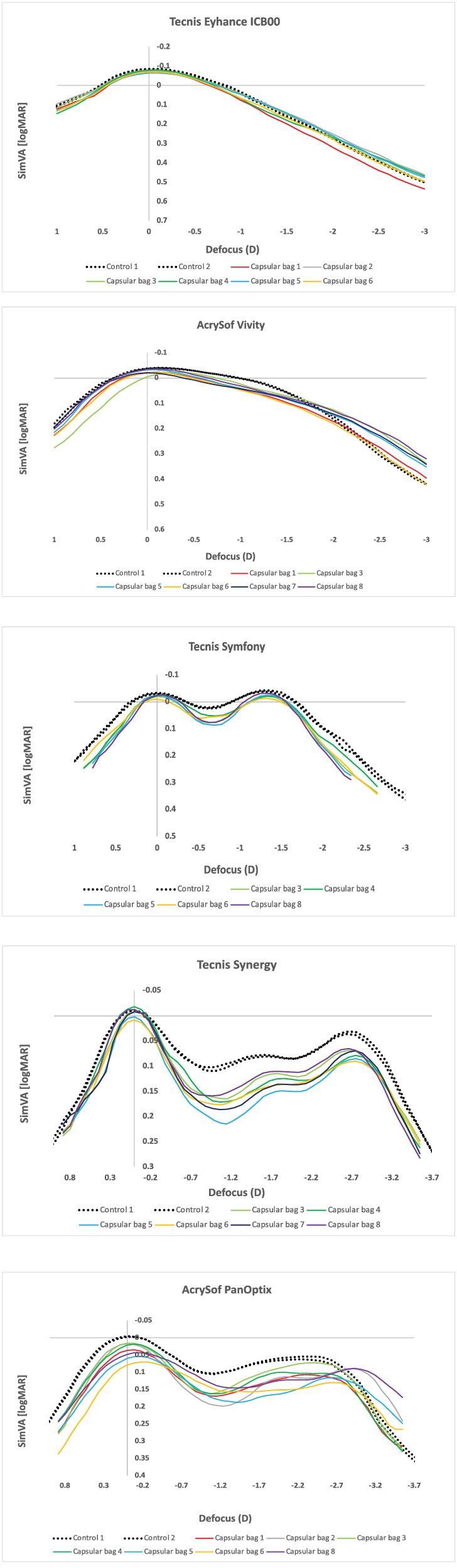
Simulated visual acuity (SimVA) in logMAR at 3.0 mm pupil size. *Solid lines* represent the IOL measured within the capsular bags, whereas the *dashed lines* show the same IOL measured prior to implantation (control measurements).

Figure 7 shows the change in SimVA with defocus for the five IOLs implanted in the capsular bag compared to their respective control measurements at a 3.0 mm pupil. All corresponding values are summarized in Table 2. The associated MTFa results are provided in Supplementary Figure S1.

**Table 2.** Simulated Visual Acuity (SimVA, logMAR) of the Five IOLs Implanted in the Capsular Bag at Far (0 D), Intermediate (−1.5 D), and Near (−2.5 D) Distances Compared to Their Respective Control Measurements at a 3.0 mm Pupil

	Far (0 D)	Intermediate (67 cm, −1.5 D)	Near (40 cm, −2.5 D)
Tecnis Eyhance ICB00,			
Mean ± SD	−0.07 ± 0.01	0.16 ± 0.02	0.39 ± 0.03
[Range]	[−0.08 to −0.06]	[0.14 to 0.20]	[0.36 to 0.44]
Control	−0.08	0.16	0.40
AcrySof Vivity,			
Mean ± SD	−0.02 ± 0.01	0.09 ± 0.01	0.24 ± 0.03
[Range]	[−0.04 to −0.01]	[0.07 to 0.10]	[0.21 to 0.29]
Control	−0.04	0.06	0.30
Tecnis Symfony,			
Mean ± SD	−0.02 ± 0.01	−0.01 ± 0.01	0.28 ± 0.02
[Range]	[−0.03 to −0.01]	[−0.02 to 0.00]	[0.26 to 0.30]
Control	−0.03	−0.03	0.23
Tecnis Synergy,			
Mean ± SD	−0.006 ± 0.01	0.15 ± 0.02	0.09 ± 0.01
[Range]	[−0.02 to 0.01]	[0.12 to 0.17]	[0.07 to 0.10]
Control	−0.01	0.08	0.04
AcrySof PanOpti,			
Mean ± SD	0.04 ± 0.02	0.15 ± 0.02	0.11 ± 0.02
[Range]	[0.02 to 0.08]	[0.12 to 0.19]	[0.07 to 0.13]
Control	−0.00	0.08	0.06

At the far focus (0 D), Tecnis Eyhance achieved −0.07 ± 0.01 [−0.08 to −0.06] logMAR, whereas AcrySof Vivity yielded slightly lower values of −0.02 ± 0.01 [−0.04 to −0.01] logMAR. The diffractive EDoF Tecnis Symfony demonstrated −0.02 ± 0.01 [−0.03 to −0.01] logMAR, Tecnis Synergy −0.01 ± 0.01 [−0.02 to 0.01] logMAR, and AcrySof PanOptix 0.04 ± 0.02 [0.02, 0.08] logMAR.

At the intermediate focus (67 cm, −1.5 D), Tecnis Eyhance reached 0.16 ± 0.02 [0.14, 0.20] logMAR. AcrySof Vivity performed better, consistent with its EDoF design, with 0.09 ± 0.01 [0.07, 0.10] logMAR, whereas Tecnis Symfony provided the best intermediate outcome with −0.01 ± 0.01 [−0.02 to 0.00] logMAR. By contrast, the trifocal lenses showed lower values: Tecnis Synergy 0.15 ± 0.02 [0.12, 0.17] logMAR, and AcrySof PanOptix 0.15 ± 0.02 [0.12, 0.19] logMAR.

At the near focus (40 cm, −2.5 D), Tecnis Eyhance measured 0.39 ± 0.03 [0.36, 0.44] logMAR. Both AcrySof Vivity (0.24 ± 0.03 [0.21, 0.29] logMAR) and Tecnis Symfony (0.28 ± 0.02 [0.26, 0.30] logMAR) performed better at this distance. The trifocal IOLs showed by far the best values, with Tecnis Synergy at 0.09 ± 0.01 [0.07, 0.10] logMAR and AcrySof PanOptix at 0.11 ± 0.02 [0.07, 0.13] logMAR.

As shown in the curves, the intermediate and near focus values of the trifocal IOLs (Tecnis Synergy and AcrySof PanOptix) were most affected by implantation in the capsular bag compared with their control measurements.

The SimVA curves for a 4.5 mm pupil are provided in Supplementary Figure S2.

### United States Air Force Test – Target Images

**Figure 8.**
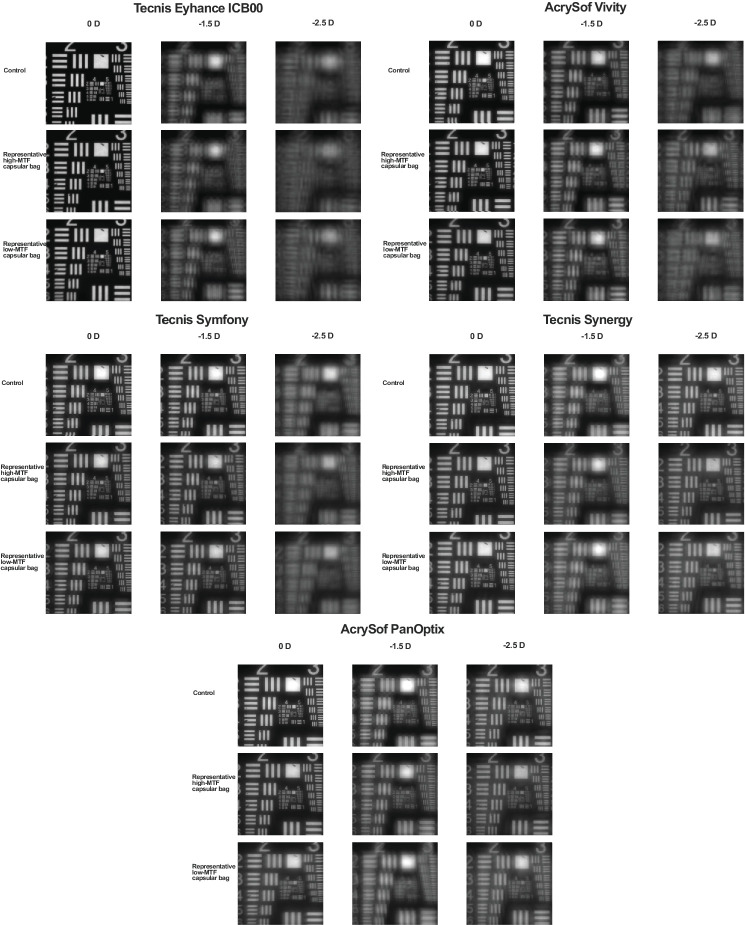
USAF target images of the 5 IOLs recorded at 3.0 mm pupil size for far, intermediate, and near focal points. The figure shows the control measurement alongside a representative capsular bag with the highest and one with the lowest measured performance, to illustrate the observed range of outcomes.

The resolution test images presented in Figure 8 support the SimVA findings. The monofocal-plus IOL (Tecnis Eyhance) demonstrated the highest optical quality at far focus, the EDoF IOLs (AcrySof Vivity and Tecnis Symfony) at intermediate focus, and the trifocal IOLs (Tecnis Synergy and AcrySof PanOptix) at near focus. This performance pattern was also preserved after implantation in the capsular bag.

**Figure 9.**
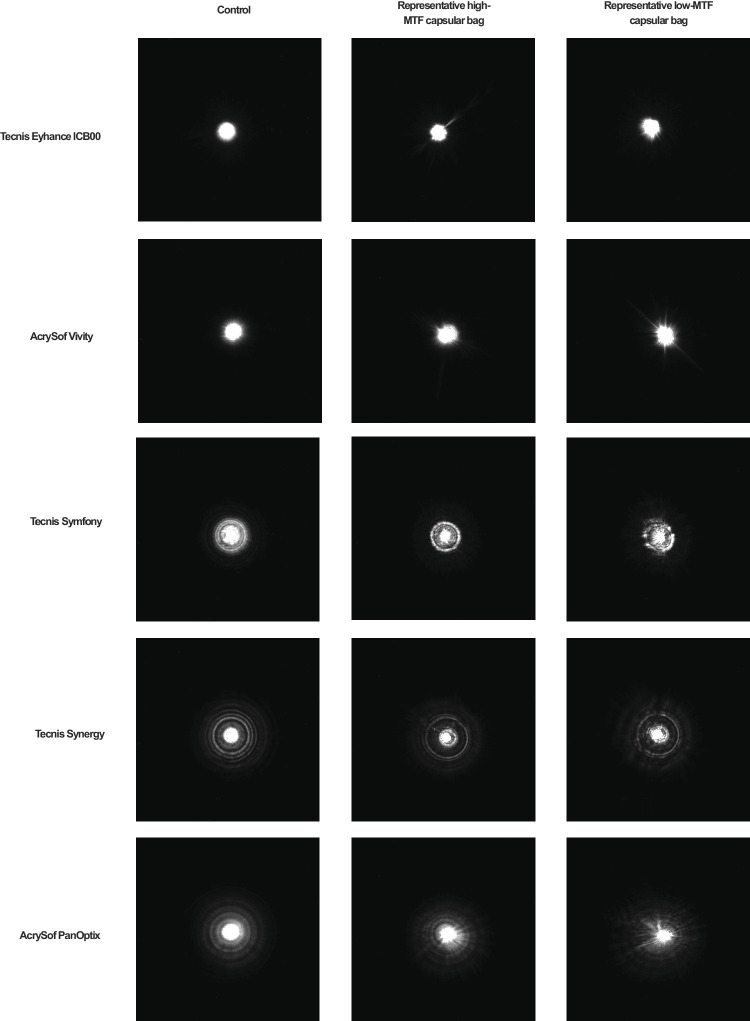
Oversaturated PSF images at a 4.5 mm aperture, visualizing the light distribution and halo patterns of the examined IOLs.

The USAF target images for a 4.5 mm pupil are provided in Supplementary Figure S3.

### PSF-Based Simulation of Photic Phenomena

An oversaturated condition was applied to visualize the light spread beyond the PSF core, which contains light of much lower intensity than the central peak. The results are presented in Figure 9. The PSF projections revealed a comparable halo profile for the monofocal-plus IOL (Tecnis Eyhance) and the refractive EDoF IOL (AcrySof Vivity). In contrast, the diffractive EDoF IOL (Tecnis Symfony) and the trifocal IOLs (Tecnis Synergy and AcrySof PanOptix) exhibited markedly more pronounced light spread, characterized by multiple concentric halo rings of increased intensity surrounding the PSF center. This pattern remained consistent after implantation in the capsular bag; however, compared with the control measurements, IOLs implanted in the capsular bag generated more irregular photic phenomena, with distorted halo ring profiles.

## Discussion

In this study, we established an ex vivo capsular bag model to investigate the optical properties of the human capsular bag and its influence on IOL performance. Over the past 2 decades, several capsular bag models have been developed, primarily to study the dynamics of posterior capsule opacification in cell culture systems. Some of these models include only the isolated capsular bag, separated from the ciliary body,^18^^,^^37^ whereas others preserve the entire choroid–ciliary body–capsular bag complex to maintain zonular support, as in vivo.^15^^–^^17^ However, existing models typically rely on bulky fixation methods, such as entomological pins or rigid PMMA/silicone rings, which are incompatible with high-precision optical bench testing.

To overcome these limitations, we developed a model consisting of two custom-designed 3D-printed rings, sutured together, that ensures an optimal size and thickness for stable handling while allowing integration into optical testing setups. This approach provides a new tool for the comprehensive preclinical evaluation of advanced IOL designs within their designated anatomic setting.

To our knowledge, straylight originating from phakic eyes’ capsular bags, has not been previously quantified in log(s). We therefore place our findings in the context of reported clinical values and measurements of explanted capsular bags from pseudophakic donor eyes with posterior capsule opacification (PCO). In our model, we measured a straylight level of 0.75 ± 0.21 [0.41, 0.92] log(s), corresponding to 6.17 ± 2.42 [2.54, 8.27] deg^2^/sr. For comparison, the straylight level of a young healthy eye has been reported as 0.9 log(s) (s = 7.9 deg^2^/sr). With aging, straylight approximately doubles, reaching 1.2 log(s) (s = 15.8 deg^2^/sr) at approximately 65 years of age, whereas cataractous eyes typically exhibit values of 1.52 log(s) (s = 33.1 deg^2^/sr).^38^ Secondary cataracts can present even higher values: in regeneratory PCO, average precapsulotomy log(s) values of 1.74 (typical cases), and 1.55 (atypical cases) have been reported, whereas in fibrotic PCO, average values of 1.24 (typical cases) and 1.31 (atypical cases) are observed.^39^ Measurements of explanted pseudophakic capsular bags alone yield lower values, averaging 0.6 [0.1, 1.1] log(s) in posterior capsule areas classified as clear, 1.2 [0.7, 1.4] log(s) in fibrotic PCO areas and 1.2 [0.6, 2.0] log(s) in regeneratory PCO areas.^40^ This difference likely reflects the fact that in vitro measurements isolate the straylight caused by the capsular bag itself, whereas in vivo measurements capture the total intraocular straylight, which additionally includes contributions from other ocular structures such as the cornea, vitreous, iris, sclera, and fundus pigmentation.

Looking at the simulated defocus curves, we can observe variability in IOL performance across different capsular bag conditions, which may help explain part of the interpatient variability in clinical outcomes following IOL implantation. For instance, defocus curve measurements in clinical settings often exhibit relatively high standard deviations, in contrast to the typically narrow ranges observed under controlled in vitro conditions. A recent meta-analysis of 31 studies on patient-reported outcomes following Tecnis Eyhance implantation demonstrated standard deviations of up to 0.13 logMAR at distance, 0.31 logMAR at intermediate, and 0.45 logMAR at near focus.^41^ Reported clinical outcomes in the meta-analysis show mean corrected distance visual acuity (CDVA) values between −0.15 and 0.05 logMAR, distance-corrected intermediate visual acuity (DCIVA) between 0.07 and 0.30 logMAR, and distance-corrected near visual acuity (DCNVA) between 0.25 and 0.55 logMAR. These ranges are in good agreement with the trends observed in our ex vivo optical measurements.

Our results further demonstrated concordance with reported patient outcomes for the EDoFs AcrySof Vivity^42^^–^^44^ and Tecnis Symfony,^45^^,^^46^ as well as the trifocal IOLs Tecnis Synergy^47^^,^^48^ and AcrySof PanOptix.^45^^,^^46^^,^^49^ For instance, Pastor-Pascual et al.^42^ reported in a cohort of 47 eyes, implanted with the AcrySof Vivity IOL, mean values of −0.02 ± 0.08 logMAR for CDVA, 0.14 ± 0.09 logMAR for DCIVA, and 0.23 ± 0.12 logMAR for DCNVA at 3 months postoperatively. Comparable results were described by Elvira et al.^43^ in 22 patients implanted with the same refractive EDoF IOL, reporting postoperative CDVA, DCIVA, and DCNVA values of 0.02 ± 0.08, 0.16 ± 0.11, and 0.26 ± 0.15 logMAR, respectively. For the Tecnis Symfony IOL, Ruiz-Mesa et al.^45^ reported mean values of −0.02 ± 0.03 logMAR (CDVA), 0.05 ± 0.04 logMAR (DCIVA), and 0.20 ± 0.07 logMAR (DCNVA), whereas Pedrotti et al.^46^ observed −0.09 ± 0.05, 0.07 ± 0.03, and 0.26 ± 0.05 logMAR for CDVA, DCIVA, and DCNVA, respectively. For the Tecnis Synergy IOL, Waring et al.^48^ reported mean CDVA, DCIVA, and DCNVA values of −0.02 ± 0.09, 0.23 ± 0.06, and 0.07 ± 0.08 logMAR, respectively, at 6 months post-surgery. For the AcrySof PanOptix IOL, Kohnen et al.^49^ reported −0.05 ± 0.072 logMAR for CDVA, 0.15 ± 0.144 logMAR for DCIVA, and 0.05 ± 0.095 logMAR for DCNVA at 3 months postoperatively.

The recorded USAF target images qualitatively confirmed the patterns observed in the MTF and SimVA analyses. Notably, trifocal IOLs appeared particularly susceptible to imperfections within the capsular bag, underlining their dependence on optimal ocular conditions for best performance. For example, PanOptix demonstrated reduced distance vision in some samples with variable responses to defocus, whereas Synergy exhibited decreased intermediate and near visual acuity compared with control measurements. These findings underscore the importance of careful patient selection for implantation of complex multifocal optics and highlight the potential of our ex vivo model to identify IOL designs that are particularly sensitive to capsular bag irregularities.

An illustrative example of this sensitivity is shown in Figure 6 for the Tecnis Synergy and Tecnis Symfony IOLs, where one capsular bag–IOL complex exhibits slightly lower MTF values at a 3.0-mm aperture than at 4.5 mm. Although this finding appears counterintuitive – because larger apertures are typically associated with increased aberrations in pseudophakic eyes – it can be explained by the presence of a localized optical disturbance within the central region of the capsular bag–IOL complex. Any microfolds or irregularities within the central 3.0-mm zone would degrade image quality when the aperture is small, as the ratio of affected to unaffected areas is relatively large. When the peripheral part of the capsular bag–IOL complex extending to 4.5 mm remains optically regular, this ratio declines as the total undisturbed area increases, thereby reducing the relative impact of the central disturbance and leading to an apparent improvement in the optical metrics. Another factor contributing to a good image quality of the Tecnis Synergy and Tecnis Symfony IOLs at a 4.5-mm aperture is their asphericity, which is optimized to compensate the human‑eye corneal aberration. The corneal model used in this study represents the average level of corneal spherical aberration.^28^ Consequently, given that these lenses are able to compensate nearly all of the spherical aberration of our model eye, their good performance at a 4.5-mm aperture under the studied conditions is to be expected. Importantly, the capsular bag in question did not exhibit any visible changes under surgical microscopy that would have justified its exclusion. Rather, it represents variability that may naturally occur within the capsular-bag complex, and capturing such optical variability was a key objective of the present study.

Although a close correspondence between simulated and clinical defocus curves was observed, it is important to note that optical bench analysis can only approximate in vivo performance and represents the “average” eye rather than individual patients. Differences between our findings and clinical outcomes may arise from several factors, including differences in clinical testing resolution, measurement variability, variations in testing distances for intermediate and near vision, and the fact that most clinical data are derived from binocular assessments, which reflects the combined effect of both eyes compared with “monocular” measurements.

Photic phenomena surrounding a light source typically result from the simultaneous projection of multiple foci by an IOL. In our study, a markedly greater light spread and more pronounced halo rings were observed for the diffractive EDoF IOL (Tecnis Symfony) and the two trifocal IOLs (Tecnis Synergy and AcrySof PanOptix), compared with the monofocal-plus IOL (Tecnis Eyhance) and the refractive EDoF IOL (AcrySof Vivity). These results align with previous reports,^30^^,^^32^^,^^50^^,^^51^ confirming that these diffractive designs, whereas offering improved intermediate and near vision, are more prone to photic disturbances.^30^^,^^32^ However, beyond these optical design-driven effects, the subjective perception of such phenomena, varies widely between patients and is strongly influenced by individual neuroadaptation processes.^52^

With our custom experimental setup, which enables precise quantification of optical parameters, we introduce a platform with multiple potential applications. A particularly promising future direction is the evaluation of PCO, using MTF, PSF, and SimVA. Although numerous in vitro studies have investigated the influence of IOL materials and designs on PCO, none have addressed the impact of PCO type and grade on the performance of different optical systems. A longitudinal assessment of how optical quality, and thus predicted visual acuity, across IOL designs is affected by the progression and morphology of PCO will offer valuable insights to guide future IOL development and optimization.

Longitudinal investigation of IOL/capsular bag interactions has previously been addressed by Wormstone et al.^53^ using a human capsular bag model originally developed by Cleary et al.^17^ and further refined by Eldred et al.^54^^,^^55^ The capsular bag and surrounding ciliary tissue are secured to a silicone ring and maintained in graded culture for 84 days. This system has provided insights into biomechanical aspects of IOL performance over time, including capsular bag contraction, angle of contact, haptic stability, and fusion footprint, thereby highlighting IOL stability as a key determinant of long-term outcomes. Light scatter was quantified at the end point using an image analysis method introduced by Eldred at al.^54^ Our model is not intended to replace these biomechanical approaches but rather to complement them by adding a quantitative optical dimension, while simultaneously enabling assessment of the aforementioned biomechanical parameters. In contrast to silicone ring-based systems, the compact and modular 3D-printed mounting used in our setup can be readily adapted to high-precision optical measurement devices through redesign of the mounting geometry. Whereas component fabrication and assembly within this model are technically more demanding than pin-based fixation in silicone rings, a major advantage is the ability to directly derive established optical quality metrics, including SimVA, that can be related to clinically meaningful end points. Future work could combine established graded culture capsular bag models with our optically integrated setup, enabling repeated assessments and dynamic evaluation of changes in optical performance over time, and thereby strengthening the translational link between capsular bag biology, IOL design, and functional visual quality.

### Limitations

A methodological limitation of the present ex vivo model is that not all five IOLs could be implanted and measured within every capsular bag as reusability is limited due to its biological constraints. Repeated implantation and explantation imposes mechanical stress on the zonular fibers and capsular bag, which can ultimately lead to instability or tearing. As previously reported for this preparation technique by Son et al.,^19^ isolated capsular structures are typically not sufficiently stable to tolerate more than three to five IOL implantations. Consequently, the number of IOLs tested per capsular bag varied, and not all bag–IOL combinations could be assessed. To minimize bias, the order of IOL implantation within each capsular bag was randomized. Preparations exhibiting marked capsular irregularities, such as prominent capsular folds arising after IOL implantation, were excluded from further analysis to avoid confounding the optical measurements.

## Conclusions

This study provides a platform for quantitative evaluation of the optical quality of IOLs within explanted human capsular bags, using the surrogate parameter SimVA. Whereas in vitro optical bench testing remains a fast, cost-effective, and reproducible standard for assessing new IOL designs, the incorporation of an ex vivo capsular bag model establishes a stronger link to postoperative vision. This approach allows evaluation of lens optical performance within its designated anatomic setting, offering valuable predictive insights into optical quality and visual outcomes before initiating clinical trials.

## Supplementary Material

Supplement 1
