# Supplementary material for: The Effect of the Capsular Bag on the Optical Performance of an IOL Measured in an Ex Vivo Model
Source: Invest Ophthalmol Vis Sci. 2026 Mar 19;67(3):46. doi: 10.1167/iovs.67.3.46 (PMC13012194; doi:10.1167/iovs.67.3.46)
Supplement: Supplement 1 [file iovs-67-3-46_s001.pdf]

Supplement

Number of eyes used	Age	Sex	Ethnicity	Time interval from death to enucleation	Method of preservation	Ocular disease
2	73	female	Caucasian	< 12h	Moist chamber	None
2	61	female	Caucasian	< 12h	Moist chamber	None
1	56	male	Caucasian	< 12h	Moist chamber	None
1	64	female	Caucasian	< 12h	Moist chamber	None
1	79	male	Caucasian	< 12h	Moist chamber	None
1	53	male	Caucasian	< 12h	Moist chamber	None
*1	72	male	Caucasian	< 12h	Moist chamber	None
*1	39	female	Caucasian	< 12h	Moist chamber	None
*1	78	female	Caucasian	< 12h	Moist chamber	None

Supplementary Table S1. Characteristics of the human donor eyes. Eyes marked with an asterisk (*) were used exclusively for feasibility testing aimed at establishing and optimizing the surgical preparation, fixation, and measurement protocol and were therefore not included in the final study dataset.

Capsular bag	Implanted IOLs
Bag 1	Tecnis Eyhance ICB00, AcrySof Vivity, AcrySof PanOptix
Bag 2	Tecnis Eyhance ICB00, AcrySof PanOptix
Bag 3	Tecnis Eyhance ICB00, AcrySof Vivity, Tecnis Symfony, Tecnis Synergy, AcrySof PanOptix
Bag 4	Tecnis Eyhance ICB00, Tecnis Symfony, Tecnis Synergy, AcrySof PanOptix
Bag 5	Tecnis Eyhance ICB00, AcrySof Vivity, Tecnis Symfony, Tecnis Synergy, AcrySof PanOptix
Bag 6	Tecnis Eyhance ICB00, AcrySof Vivity, Tecnis Symfony, Tecnis Synergy, AcrySof PanOptix
Bag 7	AcrySof Vivity, Tecnis Synergy
Bag 8	AcrySof Vivity, Tecnis Symfony, Tecnis Synergy, AcrySof PanOptix

Supplementary Table S2. Capsular bag-IOL combinations included in the analysis

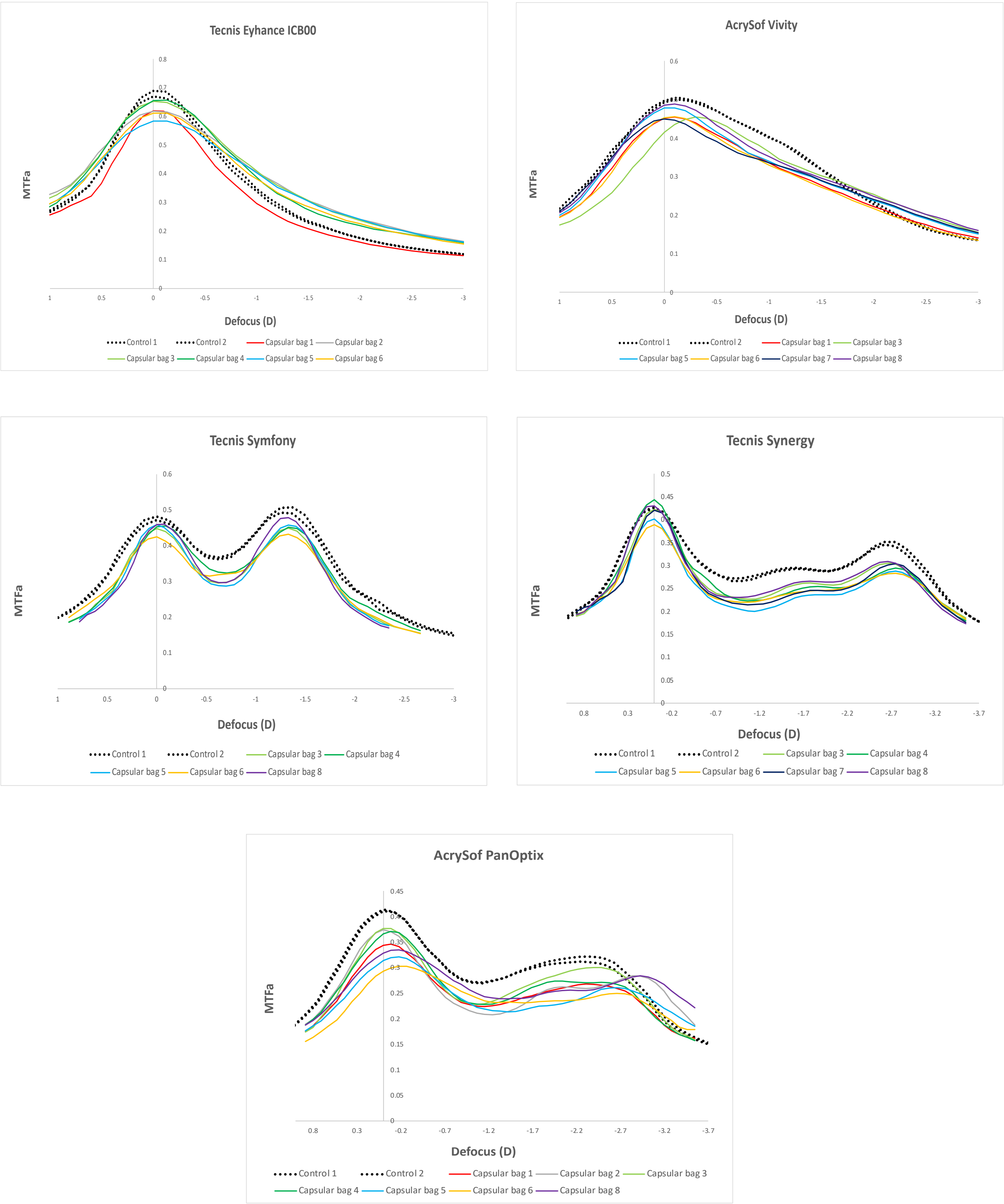

Supplementary Figure S1. Area under the MTF curve (MTFa) at 3.0 mm pupil size. Solid lines represent the IOL measured within the capsular bags, while the dashed lines show the same IOL measured prior to implantation (control measurements).

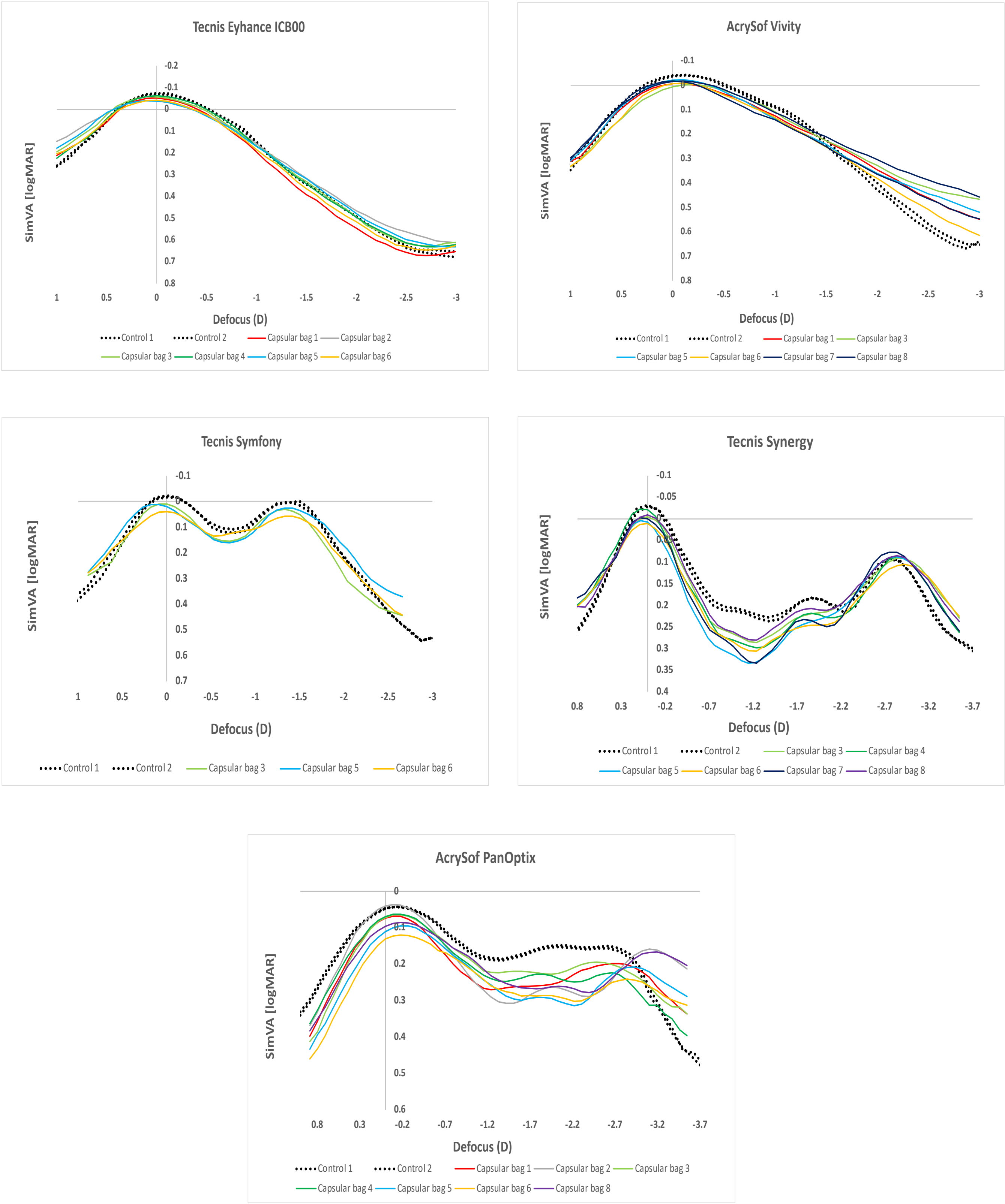

Supplementary Figure S2. Simulated visual acuity (SimVA) in logMAR at 4.5 mm pupil size. Solid lines represent the IOL measured within the capsular bags, while the dashed lines show the same IOL measured prior to implantation (control measurements).

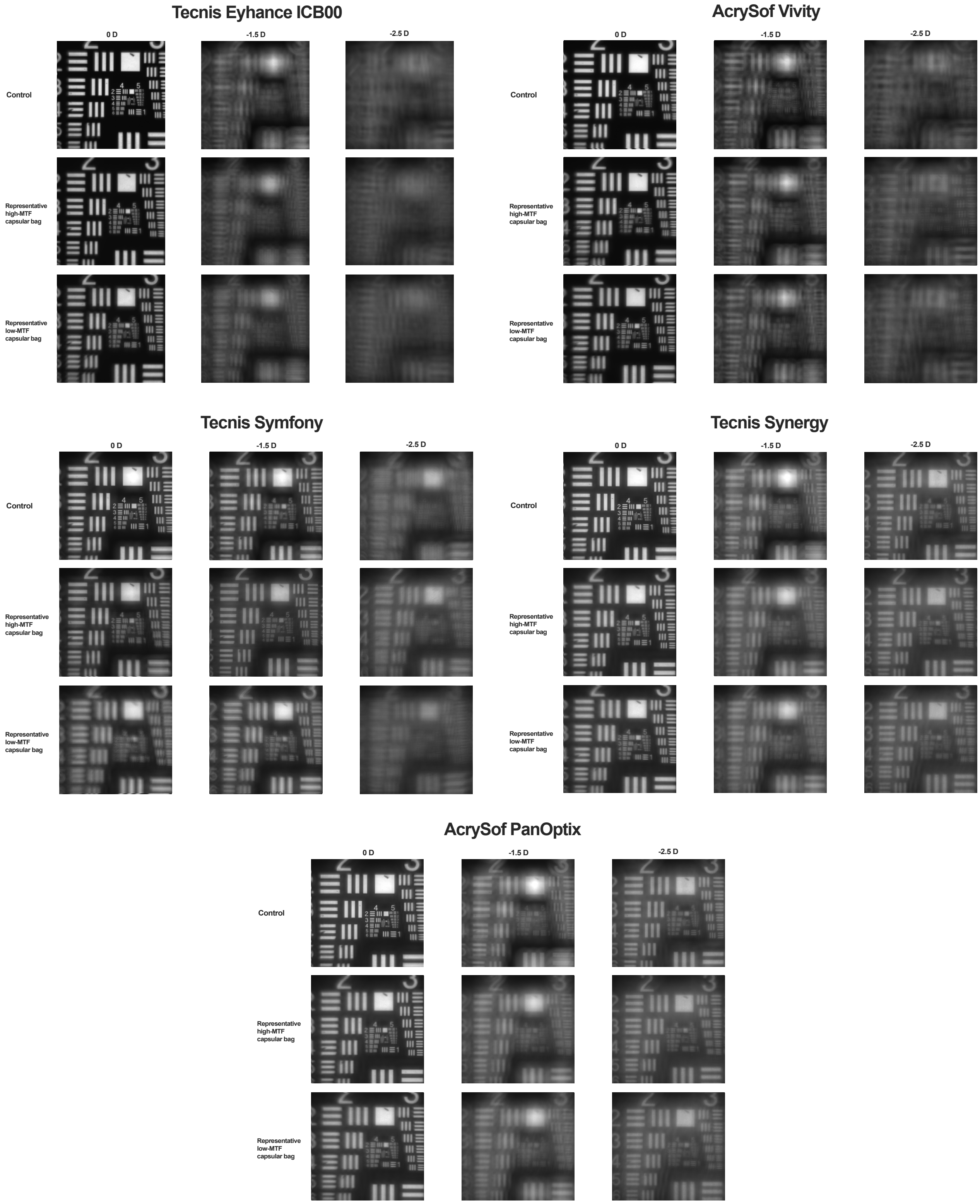

Supplementary Figure S3. USAF target images of the 5 IOLs recorded at 4.5 mm pupil size for far, intermediate and near focal points. The figure shows the control measurement alongside a representative capsular bag with the highest and one with the lowest measured performance, to illustrate the observed range of outcomes.
